# Dysfunctional telomeres in primary cells from Fanconi anemia FANCD2 patients

**DOI:** 10.1186/2041-9414-3-6

**Published:** 2012-09-14

**Authors:** Ivana Joksic, Dragana Vujic, Marija Guc-Scekic, Andreja Leskovac, Sandra Petrovic, Maryam Ojani, Juan P Trujillo, Jordi Surralles, Maja Zivkovic, Aleksandra Stankovic, Predrag Slijepcevic, Gordana Joksic

**Affiliations:** 1 Vinca Institute of Nuclear Sciences, University of Belgrade, Belgrade, Serbia; 2 Hospital of Gynecology and obstetrics “Narodni front”, Belgrade, Serbia; 3 School of Medicine, University of Belgrade, Belgrade, Serbia; 4 Mother and Child Health Care Institute of Serbia “Dr Vukan Cupic”, Belgrade, Serbia; 5 Faculty of Biology, University of Belgrade, Belgrade, Serbia; 6 Brunel University, West London, United Kingdom; 7 Department of Genetics and Microbiology, Universitat Autònoma de Barcelona, and Center for Biomedical Network Research on Rare Diseases (CIBERER), Bellaterra, Barcelona, Spain

**Keywords:** Primary FA cells, Telomere dysfunction, Expression of TRF1 and TRF2

## Abstract

**Background:**

Fanconi anemia (FA) is characterized by sensitivity to DNA cross-linking agents, mild cellular, and marked clinical radio sensitivity. In this study we investigated telomeric abnormalities of non-immortalized primary cells (lymphocytes and fibroblasts) derived from FA patients of the FA-D2 complementation group, which provides a more accurate physiological assessment than is possible with transformed cells or animal models.

**Results:**

We analyzed telomere length, telomere dysfunction-induced foci (TIFs), sister chromatid exchanges (SCE), telomere sister chromatid exchanges (T-SCE), apoptosis and expression of shelterin components TRF1 and TRF2. FANCD2 lymphocytes exhibited multiple types of telomeric abnormalities, including premature telomere shortening, increase in telomeric recombination and aberrant telomeric structures ranging from fragile to long-string extended telomeres. The baseline incidence of SCE in FANCD2 lymphocytes was reduced when compared to control, but in response to diepoxybutane (DEB) the 2-fold higher rate of SCE was observed. In contrast, control lymphocytes showed decreased SCE incidence in response to DEB treatment. FANCD2 fibroblasts revealed a high percentage of TIFs, decreased expression of TRF1 and invariable expression of TRF2. The percentage of TIFs inversely correlated with telomere length, emphasizing that telomere shortening is the major reason for the loss of telomere capping function. Upon irradiation, a significant decrease of TIFs was observed at all recovery times. Surprisingly, a considerable percentage of TIF positive cells disappeared at the same time when incidence of γ-H2AX foci was maximal. Both FANCD2 leucocytes and fibroblasts appeared to die spontaneously at higher rate than control. This trend was more evident upon irradiation; the percentage of leucocytes underwent apoptosis was 2.59- fold higher than that in control, while fibroblasts exhibited a 2- h delay before entering apoptosis.

**Conclusion:**

The results of our study showed that primary cells originating from FA-D2 patients display shorten telomeres, elevated incidence of T-SCEs and high frequency of TIFs. Disappearance of TIFs in early response to irradiation represent distinctive feature of FANCD2 cells that should be examined further.

## Background

Located at the ends of chromosomes, telomeres protect chromosomal termini from nucleolytic degradation and erroneous DNA repair; they also prevent activation of DNA damage checkpoints. Human telomeres consist of tandem arrays of a short repetitive DNA sequence (TTAGGG) oriented 5' to 3' towards the chromosome and ending in a single-stranded G rich 3’ overhang. In human cells, the size of telomeric DNA is genetically regulated, as they vary between individuals (e.g., from 10 to 15 kb in newborns). Telomere length is maintained by dynamic lengthening and shortening. Shortening results from nucleolytic degradation and incomplete DNA replication, whereas lengthening primarily results from telomerase activity, which restores telomeric sequences lost during DNA replication [1]. Telomeric repeats act as binding sites for shelterin: a six-subunit protein complex that protects chromosome ends [2]. Three shelterin subunits, TRF1, TRF2 and POT1, directly recognize TAAGGG repeats. TRF1 and TRF2 bind to double-stranded repeats of the telomere sequence, while POT1 binds single-stranded sites. TRF1 negatively regulates telomere length [3,4] but facilitates the replication of telomeres in S-phase [5]. TRF2 protects chromosome ends by repressing DNA damage induced signaling, non-homologous end joining (NHEJ) and homologous recombination repair (HRR) [6-8]. Telomere dysfunction occurs *via* the loss of telomere capping function or critical telomere shortening. Both mechanisms lead to the recruitment of DNA damage response (DDR) proteins and formation of telomere dysfunction-induced foci (TIFs) [9-11]. Defects in several DDR factors lead to telomere dysfunction in humans and mice [12]. Fanconi anemia (FA) is characterized by sensitivity to DNA cross-linking agents, mild cellular radio sensitivity and marked clinical radio sensitivity. Dysfunctional telomeres could account for this mild cellular radio sensitivity as a source of endogenous DNA damage. It is already known that FA cells show altered telomere maintenance [13-16], defects in DDR [17] and significant delay in repair kinetics of radiation-induced lesions [18]. We analyzed several telomere maintenance parameters, including telomere length, TIFs and recombination frequencies in the whole genome (sister chromatid exchange (SCE)) as well as at telomeres (telomere sister chromatid exchange (T-SCE)). In addition, we measured apoptosis and expression of the shelterin components TRF1 and TRF2 following exposure to ionizing radiation and the cross-linking agent diepoxybutane (DEB). The study was performed using non-immortalized primary cells derived from FA patients of the FANCD2 complementation group, providing a more accurate physiological assessment than is possible with transformed cells or animal models.

## Results

### Assignment of FA patients to complementation group D2

**Figure 1:**
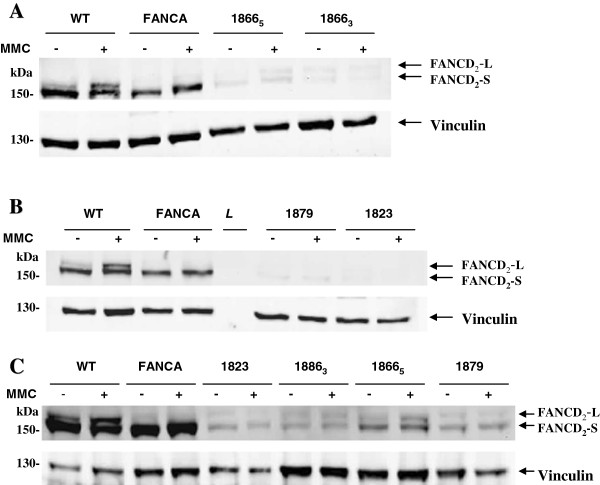
**FANCD2 immunoblotting of fibroblast extracts from FA-D2 patients and control cell lines.** Exposure of the 1823, 1866 (2 cell lines from the same patient) and 1879 patient cell lines to 50nM MMC for 24 h and subsequent analysis by Western blot (loading control Vinculin, **L:** lane for the Ladder). **A** Assignment to group FA-D2 of 1866_3_ and 1866_5_ patient cell lines on the basis of the absence FANCD2 on Western blot. **B** Assignment to group FA-D2 of 1823 and 1879 patient cell lines on the basis of the absence FANCD2 band on Western blot. **C** Over-exposure of immunoblots reveals that 1823, 1866_3_, 1866_5_ and 1879 patient cell lines show faint but visible FANCD2 bands in response to MMC.

FA patients were assigned to FA-D2 subtype by immunoblotting. The absence of FANCD2 bands on standard exposure immunoblots suggested that all three patients (1823, 1866 and 1879) belong to complementation group FA-D2 (Figure 1). The over-exposure of the films showed faint FANCD2 bands upon MMC treatment unequivocally indicating that all patients have hypomorphic mutations in FANCD2 as previously described for a larger population of FA-D2 patients [19]. For patient 2093, complementation group analysis was previously performed (Universität Würzburg, Institut für Humangenetik, Germany) and showed that she also belongs to FA-D2 complementation group.

### Telomere length analysis in FANCD2 lymphocytes by quantitative fluorescence *in situ* hybridization

**Figure 2:**
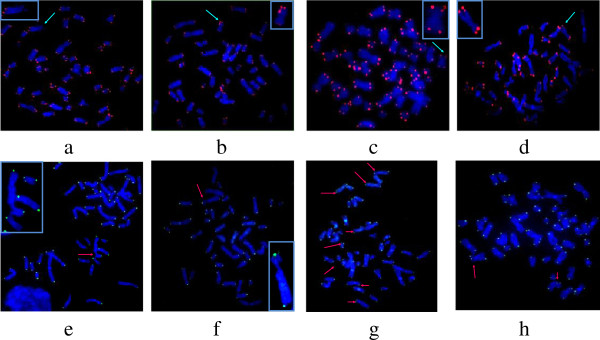
**Representative images of metaphase spreads hybridized with telomeric PNA probes using Q-FISH (panels a-d) and CO-FISH (panels e-h) protocols.(b-d)** Aberrant telomeric structures (blue arrows indicate long strings of fragmented telomeric signals: indicative of fragile telomeres) **(e-h)** The majority of chromosomes within a metaphase spread showed characteristic CO-FISH hybridization patterns in which telomere signals at opposite ends of the chromosome were in the trans position with respect to the sister chromatids. **(a,e** and **f)** Chromatid breakages in metaphase events of FANCD2 cells (arrows indicate no telomeric signals on the breakage points). **(g-h)** Telomere sister chromatid exchanges (red arrows).

**Figure 3:**
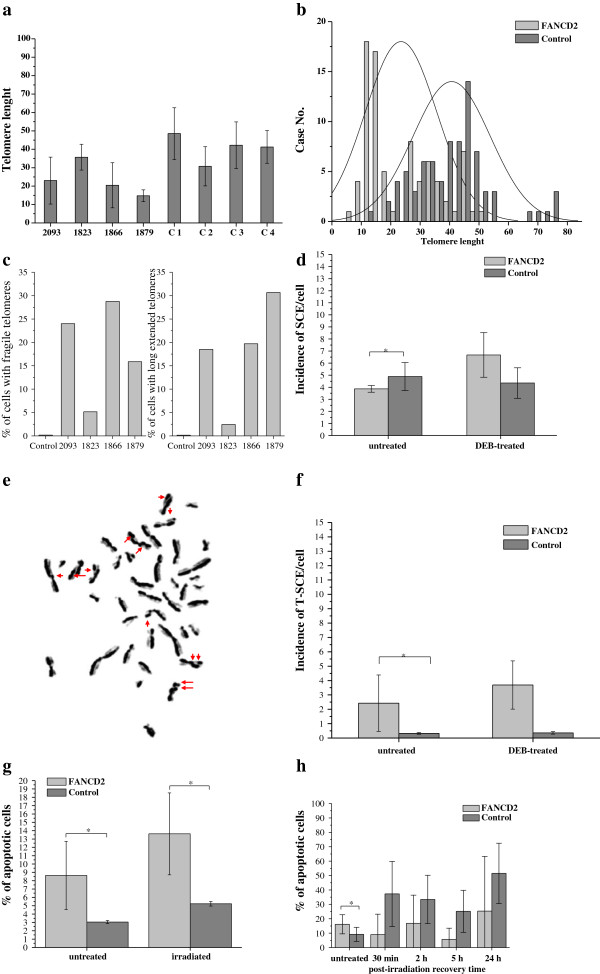
**Results of analyses in FANCD2 and control lymphocytes. a** Average telomere length (arbitrary units of RTLU ± SD) measured by Q-FISH in FANCD2 (1823, 1866, 1879 and 2093) and control (C1-C4) cells. Cells originated from FANCD2 patients display shorter telomeres than the age-matched controls (p = 0.024). **b** The distribution of telomere length indicated that control lymphocytes displayed typical Poisson distributions for individual chromosome telomere length, whereas the peak of Poisson distribution was shifted leftward in FANCD2 lymphocytes. **c** The percentage of fragile and long extended telomeres: in FANCD2 lymphocytes the percentage of cells displaying fragile telomeres is 18.45% on the average, wheras percentage of cells displaying long-extended telomeres is 17.83%. **d** SCE analysis. The incidence of spontaneous SCE in FANCD2 lymphocytes was significantly reduced when compared to control (p < 0.05). In response to DEB, almost two-fold increase of SCEs was observed in FANCD2 lymphocytes relative to the baseline state before treatment (p = 0.04). Metaphase spread showing DEB treated FANCD2 lymphocytes with 13 SCE and two chromosomes with dSCE . **f** CO-FISH analysis**.** The incidence of spontaneously occurring T-SCEs in FANCD2 lymphocytes was significantly higher than that in control cells (p = 0.026), and further increased in response to DEB. **g** Apoptosis assay showed a significant difference between FANCD2 and control leucocytes in percentage of spontaneously dying cells as well as in percentage of apoptotic cells induced by ionizing radiation. The data are presented as mean ± SD. **h** Apotosis of FANCD2 and control fibroblasts: FANCD2 cells spontaneously die with higher rate than controls and exhibit a mild delay in entering apoptosis. The data are presented as mean ± SD.

Representative examples of metaphase spreads hybridized with telomeric PNA probes using Q-FISH and CO-FISH protocols are presented in Figure 2. The measurement of telomere length (Figure 3a) revealed that FA-D2 patients exhibited reduced lymphocyte telomere length (23.50 ± 8.80), relative to age-matched controls (40.70 ± 7.36) (P = 0.024). The distribution of telomere length (Figure 3b) indicated that, in FANCD2 cells, 5% of telomeres displayed lengths of 0–10 relative length telomere unit (RTLU), and more than 50% of all telomeres were only 10–20 RTLU long. In control lymphocytes, the majority of telomeres were 30–50 RTLU long (62.5%), short telomeres were not present and 15% of telomeres displayed lengths of 50–80 RTLU. Control lymphocytes displayed typical Poisson distributions for individual chromosome telomere length, whereas the peak of Poisson distribution was shifted leftward in FANCD2 cells. The average percentage of cells displaying fragile telomeres (18,45%) and long-extended telomeres (17,83%) in FANCD2 lymphocytes are presented in Figure 3c.

### Effects of diepoxybutane on the incidence of sister chromatid exchange and telomere sister chromatid exchange in FANCD2 lymphocytes

The incidence of spontaneous SCE in FANCD2 lymphocytes (3.87 ± 0.28) was significantly reduced when compared to control (4.89 ± 1.16) (P < 0.05) (Figure 3d). In response to DEB, a nearly two-fold increase of SCEs was observed in FANCD2 lymphocytes (6.68 ± 1.85), relative to the baseline state before treatment (P = 0.04). Appearance of double SCE (dSCE) in response to DEB frequently were observed (Figure 3e). In contrast, the same concentration of DEB decreased SCEs in control cells (4.35 ± 1.27) (P = 0.034).

CO-FISH indicated that the incidence of spontaneously occurring T-SCEs in FANCD2 primary lymphocytes (2.43 ± 1.96) was significantly higher than that of control cells (P = 0.026), and further increased upon exposure to DEB (3.69 ± 1.68). In control cells, the incidence of spontaneously occurring T-SCEs was minimal (0.32 ± 0.06) and almost unchanged in response to DEB (Figure 3f).

### Effects of ionizing radiation on apoptosis of FANCD2 leucocytes and fibroblasts

As shown in Figure 3g and Figure 3h both FANCD2 leucocytes and fibroblasts appeared to die spontaneously at higher rate than control. Upon irradiation the percentage of leucocytes underwent apoptosis was 2.59-fold higher than that in control (P = 0.0001), while in fibroblasts, the mild 2-h delay of entering apoptosis was observed. Both FANCD2 and control fibroblasts extensively enter apoptosis 24 h after irradiation.

### Effects of diepoxybutane and ionizing radiation on percentage of telomere dysfunction-induced foci in FANCD2 fibroblasts

**Figure 4:**
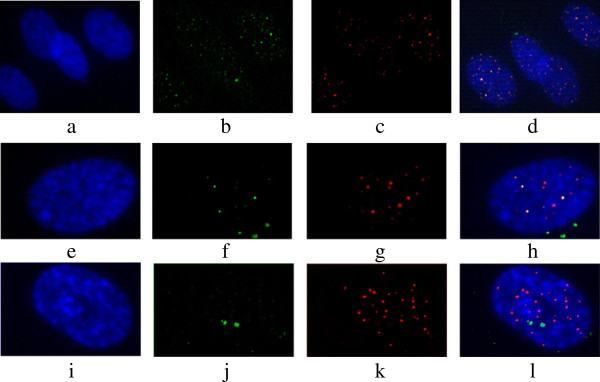
**Representative images of cell nuclei with or without telomere dysfunction-induced foci (panels a-l).** (**a-h**) Representative examples of FANCD2 nuclei with TIFs. (**a, e**) DAPI (**b, f**) γ-H2AX, (**c, g**) Telomeres and (**d, h**) Merged. When green and red signals overlap (merge) a yellow pattern is seen, indicating co-localisation of γ-H2AX with telomeres (**i-l**) Control cell nuclei with distinct γ-H2AX and telomere signals (no TIFs), (**i**) DAPI, (**j**) γ-H2AX, (**k**) Telomeres and (**l**) Merged. Zeiss-Axioimager A1 microscope equipped with a CCD camera, Axiocam image acquisition software (Imaging Associate) and software package from MetaSystem were used for analysis.

**Figure 5:**
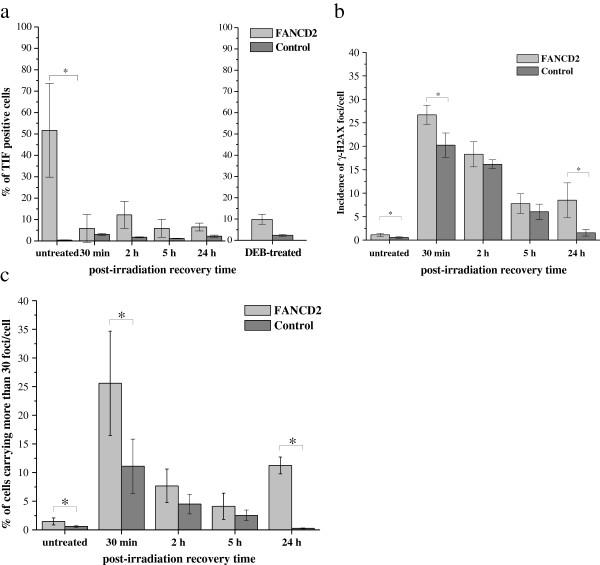
**Results of analyses in FANCD2 and control fibroblasts. a** Average incidence of radiation- induced γ-H2AX foci per cell (mean ± SD) in FANCD2 (n = 4) and control fibroblasts (n = 6). The total number of γ-H2AX foci in FANCD2 fibroblasts was significantly higher at all recovery times, relative to control samples. **b** Percentage of FANCD2 cells displaying more than 30 γ-H2AX foci per cell within recovery time of 30 min, revealed a 2-fold increase compared to that in control fibroblasts (p < 0.05). **c** TIF analysis. In FANCD2 fibroblasts, in average 51.7% of nuclei were TIF-positive (displaying more than 5 TIFs per nucleus). The level of TIFs inversely correlates with telomere length (P = 0.03). Percentage of TIF positive cells significantly decreased after irradiation. At recovery time of 30 min considerable percentage of TIF positive cells disappeared at the same time when the incidence of γ-H2AX foci was maximal. Afterwards, TIFs appeared again, but in less extent and remained almost the same at all later recovery times. 24 h after DEB treatment, the percentage of TIF-positive FANCD2 cells also was reduced (p = 0.026) when compared to a self-state before treatment.

Representative images of FANCD2 nuclei hybridized with telomeric PNA probes using TIF protocol are presented in Figure 4. The results of γ-H2AX and TIF analyses in FANCD2 and control fibroblasts are presented in Figure 5. While γ-H2AX foci were present in both control (5%) and FANCD2 fibroblasts (13.27%) enhanced co-localisation of the γ-H2AX with telomeres was observed only in FANCD2 fibroblasts. As shown in Figure 5a, in average 51.7% of γ-H2AX foci positive cells were TIF-positive (displaying more than five TIFs per nucleus). It is worth noting that the level of spontaneously occurring TIFs inversely correlated with telomere length (P = 0.03), which emphasizes that shortened telomeres are the underlying cause of their dysfunction.

After DEB treatment, the percentage of TIF-positive FANCD2 cells was significantly reduced (P = 0.026) compared to self-state before treatment, whereas in control fibroblasts the percentage of TIF-positive cells slightly increased (Figure 5a).

Upon irradiation, the total number of γ-H2AX foci in FANCD2 fibroblasts was significantly higher at all recovery times, relative to control samples (Figure 5b). Distribution of radiation-induced foci among cells with a recovery time of 30 min, revealed a 2-fold increase in the number of cells containing more than 30 foci per cell; significantly higher compared to that in control fibroblasts (P < 0.05) (Figure 5b). Unexpected, at the same recovery time (30 min) when the incidence of γ-H2AX foci was maximal significant decrease of TIFs was observed (Figure 5c). Afterwards, the percentage of TIF positive cells slightly increased and remained almost the same over the next 2, 5 and 24 h. Percentages of dying FANCD2 fibroblasts were almost the same prior and 2 h after irradiation (Figure 5d) indicating that cell loss is not the reason for TIFs disappearance. The observations that FANCD2 cells exhibit a mild 2-h delay in entry into apoptosis after irradiation and a 2-fold higher rate of spontaneous apoptosis relative to control signify difficulties in activation of physiological mechanisms of cell death that happened exactly at the time when treated cells did not display TIFs.

### Relative expression of TRF1 and TRF2 mRNAs

**Figure 6:**
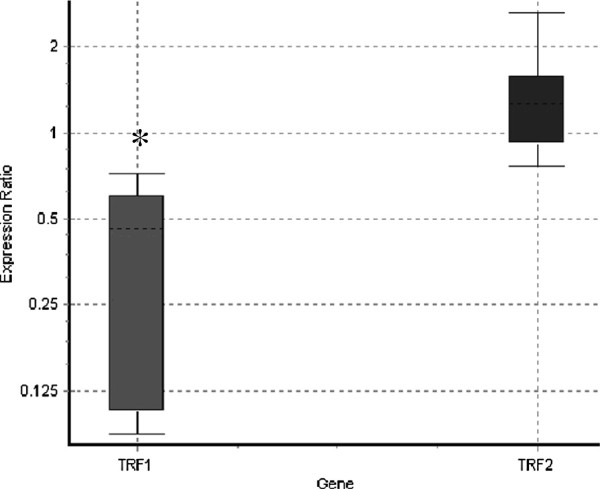
**The relative expression of shelterin compounds TRF1 and TRF2 mRNA in FANCD2 (n = 4) and control fibroblasts (n = 6).** The relative level of TRF1 and TRF2 mRNA was standardized against the housekeeping gene 18 s rRNA and calculated using relative expression software tool, REST 09. The squares represent relative expression of TFR1 and TRF2 in FANCD2 cells *versus* control cells. The box area in a whisker-box plot encompasses 50% of all observations, the dotted line represents the sample median and the whiskers represent the outer 50% of observations.

Relative to control cells, the relative expression of TRF1 mRNA in FANCD2 fibroblasts was significantly down-regulated by a mean factor of 0.31 (S.E. range 0.093 – 0.610, 95%CI 0.088-0.697, P = 0.000) (Figure 6). TRF2 gene expression was similar in both FANCD2 and control cells (mean factor 1.178, S.E. range 0.814 - 1.71, 95% CI 0.771 - 1.801, P = 0.7). The TRF1:TRF2 ratio was close to 1 (P = 0.7) in control cells. We did not detect significant differences in TRF1 or TRF2 gene expression between FANCD2 and control cells following irradiation or treatment with DEB.

## Discussion

This study provides evidence that primary cells originating from FA-D2 patients display shorter telomeres than their age-matched control counterparts, high frequency of dysfunctional telomeres, and broad spectrum of aberrant telomeric structures ranging from fragile to long-string extended telomeres. Furthermore, telomeres in FANCD2 primary cells display extra heterogeneous telomere lengths of individual chromosomes and elevated incidence of T-SCEs. The recent work of Ball et al. [13], Callen et al. [14] and Leteurtre et al. [20] have demonstrated the presence of shorter telomeres in FA patients (mostly belonging to the complementation group FA-A) than their age-matched controls, which is consistent with our findings in FA-D2 patients. Herman et al. [21] proposed that average telomere length is not the only determinant of telomere function, emphasizing that critically short individual telomeres can trigger cellular responses to a loss of telomere function. The heterogeneity of telomere length that was present in our FA-D2 patients was expressed as SD of telomere length; our correlative analysis supports their hypothesis that critically short telomeres trigger a DDR response that reduces telomere capping function. Metaphase events presented in Figure 2(b-d), illustrate a hybridization pattern with aberrant telomeric structures ranging from fragmented telomeric signals to an extended strand of telomeric DNA; these patterns are indicative of telomere dysfunction. The findings of this study support those reported in several prior studies [22-26] with embryonic stem cells lacking TRF1. In this study, all FA-D2 patients exhibited decreased TRF1 expression, which correlates to telomere defects. Because TRF1 has been described as a negative regulator of telomere length [27], reduced TRF1 expression is thought to induce telomere elongation; however, this did not occur. In contrast to tumor cells [28,29], down-regulation of TRF1 in FANCD2 cells is probably linked to the functional role of TFR1 in regulating cell cycle progression. In response to DNA damage, ATM beside histone H2AX, phosphorylates TRF1 to inhibit its mitotic function and prevent abortive mitosis. Given that FANCD2 cells exhibit a 2-fold increase in the number of cells displaying more than 30 γH2AX-foci per cell after irradiation, down-regulation of TRF1 may be a consequence of its degradation due to the failure of cells to progress into the cell cycle normally, as is the case with control cells. Down-regulation of TRF1 may also provide cells to increase their proliferative potential.

By employing the CO-FISH technique, our study demonstrates a two-fold enhancement of T-SCEs in FANCD2 cells, relative to control cells. This may result from an attempt to replenish lost telomeric sequences by homologous recombination between sister telomeres. While there is currently no direct evidence that T-SCEs modify telomere length, the recombinogenic behavior of telomeres in FANCD2-deficient cells could contribute to heterogeneity in telomere length, which in turn activates DDR. Recent work by Lyakhovich et al. [30] with immortalized FANCD2 fibroblasts has shown that extrachromosomal circular telomeric structures in FANCD2-deficient cells are not linked to telomeric recombination between sister chromatids. In our study, FISH analysis of primary FANCD2 cells did not reveal extrachromosomal telomeric signals; in contrast, fragile telomeres and split or extended telomeric signals were frequently observed. Recent work of Polanska et al. [31], have shown that chromatin associated protein (HMGB1) also maintain telomere integrity. The most interesting finding in their study is that HMGB1^−/−^ cells exhibit heterogeneous telomere lengths of individual chromosomes and highly extended telomeres, as we found in lymphocytes of our FANCD2 patients.

Considering the role of TRF2 in telomere maintenance, many studies with mouse models have revealed that T-SCE are repressed by TRF2 [32]. Expression of TRF2 in either FA cells or control cells was nearly identical, indicating that an increase of T-SCE could be attributed to some other proteins; examples of which include those that regulate histone heterochromatin density in telomeric and sub-telomeric regions and influence chromatin remodeling, as suggested by Schoeftner and Blasco [33], and Polanska et al. [31].

The novel finding of our study is that a remarkably high percentage of FANCD2 cells had dysfunctional telomeres that were mostly caused by telomere shortening collectively with extensive telomere length heterogeneity of individual chromosomes (i.e., 1866 cells displayed 74% of TIF positive cells). Although elevated incidence of γH2AX foci in FANCD2 cells vs. control was observed at all recovery times after irradiation, a significant decrease of TIFs was determined at the same post-irradiation times. Particularly unexpected was a considerable disappearance of TIF positive cells 30 min after irradiation, when the incidence of γH2AX foci was maximal (Figure 5a). Afterwards, TIFs appeared again, but in less extent and remained almost the same at all later recovery times. When we look at the apoptosis profile of FANCD2, it is evident that percentage of dying cells was almost the same up to 2 h after irradiation (compared to a baseline state), indicating that cell loss is not the reason for TIFs disappearance. The work of Kaul et al. [34] have shown that normal human cells can tolerate small number of TIFs, so called “intermediate-state” telomeres. We hypothesize that repair-deficient FANCD2 cells could possibly tolerate bigger number of intermediate-state telomeres. However, upon irradiation FANCD2 cells display fast and marked reduction of TIFs, which is likely due to a recruitment of γH2AX away from telomeres to newly formed DSBs. Irradiated FANCD2 fibroblasts exhibit mild delay of 2 h in ingoing apoptosis relative to control, signifying difficulties in activation of physiological mechanisms of cell death. Accelerated telomere shortening in radiosensitive cell lines has been reported in several prior publications [35,36]. Collectively, the data presented by our study suggests that uncapping of telomeres results from an increase in telomere recombination, in addition to accelerated telomere shortening; this is consistent with general enhancements in the activity of HR. For example, 1823 cells possess telomeres of nearly normal length and display no increase in HR activity; therefore, we conclude that only cells with sufficiently long telomeres bypass HR to replenish telomeric repeats. In FANCA cells, telomere dysfunction-induced foci were found in less than 10% of cells. FANCA also exhibit shorter telomeres than controls, but in contrast to FANCD2 exhibit fast progression, no delay in cell cycle and display very few TIFs in base-line state, and in response to irradiation behave in a similar manner as controls (unpublished data).

In response to cross-linking agents, FANCD2 lymphocytes exhibited a 2-fold increase in the frequency of SCE when compared to control cells. In control lymphocytes, progression of SCE in response to DEB was suppressed. To the best of our knowledge, this is the first report that describes a role of the cross-linking agent DEB in suppressing the occurrence of SCE in normal cells and significantly enhancing it in FA cells. Particularly frequent double SCE were observed, as illustrated in Figure 3f. Increased SCE incidence upon DEB treatment suggests that replication fork collapse may occur because of the inability of cells to repair replication-blocking lesions and initiation of homologous recombination. According to findings in our study incidence of SCE induced by DEB can be used as an adjunct diagnostic test for FA cellular phenotype. High-rates of homologous recombination in human FA cells first were observed by Thyagarajan and Campbell [37], who discovered that recombination levels in Fanconi anemia cells were nearly 100-fold higher than in control cells. The authors proposed that inappropriate increase in the amount of homologous recombination might contribute to genomic instability and cancer susceptibility that characterized FA. Rouet et al. [38] found that HR processes operate on DSBs, which can further rationalize our finding that enhanced HR activity was present in treated FA-D2 cells [38]. Work of Yamamoto et al. [39] have shown that sensitivity of FANCD2 cells to cross-linking agents and ionizing radiation is mainly due to premature termination of repair during DNA synthesis. Nakanishi and colleagues [40] provide evidence that cells derived from FANCA, FANCG and FANCD2 patients are defective, to a mild degree in HR. Smogorzewska et al. [41] verify that siRNA mediated depletion of FANCI and FANCD2 resulted in reduced HR activities. Indeed, the results of our study revealed reduced rate of SCE in FANCD2 cells and higher rate of spontaneously dying cells *versus* control, which is in accordance to previously mentioned findings.

The mechanism by which telomere dysfunction is initiated remains unresolved. Broccoli et al. [42] postulated that dysfunctional telomeres may fall below a threshold of telomere-associated proteins, such as TRF1 and TRF2, thereby impairing the formation of the telomere loop structure; however, subsequent studies, including our study, determined that this effect is not connected to these important shelterin components. Morish and Greider [43] reported that mammalian cells utilize several different non-telomerase mechanisms for telomere maintenance, which include recombination initiated by short telomeres; the results of our study support this hypothesis. The work of Fan and colleagues [44] have shown that depletion of FANCD2 is associated with decrease in recombination between telomeres. In our study the opposite results were obtained, which can be explained in part by the fact that truncated FANCD2 protein in primary FA-D2 cells is still competent to function in HR. Telomere findings in our study confirm that FANCD2 cells maintain telomeres trough homologous recombination displaying a more heterogeneous length and increased chromosomal instability including TIFs. Interestingly no fusions were observed in either lymphocytes or fibroblasts indicating that telomeres are not critically shorten to be fused. This observation is in line with work of Cesare and colleagues who postulated that not all uncapped telomeres are equal, pointing out that some cells as cancerous cells posses TIF positive telomeres that are not involved in fusions [45]. The similar distinctiveness of telomeres in primary cells originated from FA-D2 patients are observed in our study.

Taken together, our study revealed that high percentage of TIFs that characterized FANCD2 primary cells is most likely induced by critically shortened telomeres. In addition, fragile and long-string extended telomeres along with disappearance of TIFs in early response to irradiation are distinctive features of FANCD2 primary cells. Genomic instability resulting from telomere dysfunction is a plausible explanation for the increased cancer susceptibility and clinical hypersensitivity of all FA patients, not only of the FA-A complementation group.

## Methods

### Patients

The patients included in this study were four female children with Fanconi anemia belonging to complementation group FA-D2. The patients were 8 ± 5 years of age. Cellular FA phenotype was diagnosed with classic chromosomal breakage DEB tests of peripheral blood lymphocytes, as described by Auerbach et al. [46]. Patients who displayed DEB-positive lymphocyte phenotypes underwent skin biopsies to assess FA fibroblast phenotype and to determine genetic subtype. Healthy age-matched volunteers served as control groups (12 subjects as controls for SCE analysis, and 4 subjects as controls for telomere length analysis). All methods were approved by the Ethical Committee of the Mother and Child Health Care Institute of Serbia, and the subjects’ parents signed informed consent forms.

### Cell culture

#### Lymphocytes

Peripheral blood was collected from all subjects and deposited into heparinized vacutainer tubes. Lymphocytes were cultured in PBmax karyotyping medium (Invitrogen-Gibco, Paisley, UK). Adequate numbers of cell cultures per subject were established, enabling SCE analysis, T-SCE analysis and telomere length analysis *via* quantitative FISH (Q-FISH) in metaphase cells.

#### Fibroblasts

Cells were grown in DMEM (Invitrogen-Gibco, Paisley, UK) supplemented with 10% fetal bovine serum (Invitrogen-Gibco, Paisley, UK) under standard tissue culture conditions: 37°C in 10% CO_2._ Cells were harvested in duplicate and propagated to 80-90% confluence, at which point cells were subcultured. A 1.0 mL cell suspension was prepared at a density of 50,000 cells for each sample, and the cells were seeded onto polylysine-coated glass slides (Sigma Chemical Co., St Louis, MO) and incubated overnight in atmosphere-controlled chamber containing 10% CO_2._ As a control, primary cells from skin biopsies of six healthy subjects undergoing plastic surgery were also used.

#### Irradiation

Irradiation was performed with a ^60^Co γ-ray source (a 2.0 Gy dose at 0.45 Gy/min). Fibroblasts were irradiated on polyprep slides and then returned to the tissue culture incubator and examined at various recovery times (30 min, 2 h, 5 h and 24 h).

#### Diepoxybutane treatment

Cells were treated with DEB at final concentration of 0.1 μg/mL or 0.01 μg/mL for lymphocytes and fibroblasts, respectively. Fibroblasts were exposed to DEB for 24 h, whereas lymphocytes were exposed for 72 h.

### Western blot analysis for the subtyping of FA patients

Exposure of the patients and controls cell lines to 50nM mitomycin C (MMC) for 24 h and subsequently analysis by FANCD2 western blot was performed as previously described by Casado et al. [47] and Castella et al. [48]. Anti-FANCA antibodies (gift of the Fanconi Anemia Research Fund, dilution 1:500) and anti-FANCD2 antibodies (Santa Cruz Biotechnology, Santa Cruz, California, USA) (dilution 1:2500) were used. Assignment of group FA-D2 was done on the basis of the absence FANCD2 on Western blots usingWestern Breeze Immunodetection Kit (Invitrogen, Grand Island, NY, USA). The over-exposure of films by the chemiluminescence technique to identify residual FANCD2 protein levels was performed as described by Kalb et al. [19].

### Quantitative fluorescence *in situ* hybridization

For Q-FISH, cells were harvested under standard conditions. During the final 3 h of incubation, 0.1 μg/mL of colchicine (Sigma Chemical Co., St Louis, MO) was added to cell cultures. Subsequently, a hypotonic solution (5.6 g/l of KCl) was added, followed by three consecutive fixations with 3:1 methanol/acetic acid; thereafter, slides were prepared. Q-FISH was performed as described by Slijepcevic [49]. Briefly, after appropriate wash steps, the slides were hybridized with the Cy-3 labeled telomeric PNA probe (CCCTAA) 3' and left in a dark humidified chamber for 2 h. The slides were then washed in 70% formamide and stained with 4', 6’-diamidino-2-phenylindole (DAPI)-containing mounting medium (Vector Laboratories, UK). Chromosomal analysis was performed with a Ziess-Axioplan2 microscope equipped with a CCD camera, Axiocam image acquisition software (Imaging Associate) and software package from MetaSystem. Measurements were reported as arbitrary relative telomere length units (RTLU), which are defined as the ratio of signal intensity between telomeres and a centromere chromosome 2 reference signal.

### Sister chromatid exchange analysis

Cells intended for SCE analysis were cultured in duplicate and allowed to undergo two rounds of replication in the presence of 5-bromo-2-deoxyuridine (BrdU) (Sigma Chemical Co., St Louis, MO) to achieve differentiation of sister chromatids. BrdU was added one hour after culture initiation and cells were harvested at 72 h, the final three hours of which were conducted in the presence of colchicine (final concentration 0.1 μg/mL). One of these cultures was used to analyze spontaneously occurring SCE, while a second was treated with DEB one hour after culture initiation, and continued for as long as 72 h. After conventional hypotonic treatment, cells were fixed three times in 3:1 methanol: acetic acid. Afterward, the pellet was spread onto glass microscope slides. Differential staining of sister chromatids was performed *via* standard fluorescence-plus-Giemsa (FPG) technique [50]. A total of 30 complete second-division metaphase cells were analyzed per sample with a Ziess-Axioplan2 microscope.

### Chromosome orientation fluorescence *in situ* hybridization

Parallel cultures were used to conduct telomere T-SCE analysis. The chromosome orientation fluorescence *in situ* hybridization (CO-FISH) protocol of Bailey et al. [51] was performed with some modification for lymphocytes: 48 h after the start of culture, 30 μM BrdU and 10 μM bromodeoxycytidine (BrdC) (Sigma Chemical Co., St Louis, MO) were added and cells were allowed to progress through S phase and into the first mitosis the following day. Colchicine was added to a final concentration of 0.1 μg/mL during the last 3 h of culture. Preparation of cultures was performed according to the standard protocol (hypotonic treatment and three consecutive fixations in 3:1 methanol:acetic acid). Cell pellets were spread onto glass microscope slides using cytogenetic techniques. Newly synthesized DNA strands were degraded by treating fixed cells on glass microscope slides with 0.5 μg/mL Hoechst 33258 for 15 min (Sigma Chemical Co., St Louis, MO), 313 nm light for 30 min and 3 U/μL Exo III (Promega, Madison, WI) at room temperature for 10 min. Slides were washed in PBS and subsequently dehydrated in a series of ethanol washes. Tel Cy3-dUTP (20 μL), a telomere PNA probe (Panagene, Korea) was placed onto each slide, covered with glass coverslips and hybridized on a hot plate for 5 min at 80°C. After hybridization, slides were maintained in the dark in a wet compartment for 2 h. Afterwards, the slides were washed three times in 70% formamide/2xSSC for 15 min, and then washed three times in PBS for 5 min. Samples were then dehydrated in ice-cold ethanol, mounted in DAPI (Vectashield), covered with coverslips and sealed. At least 20 metaphase events from each patient were quantified by CO-FISH. Each metaphase event was analyzed with Axiocam image acquisition software (Imaging Associate) and a software package from MetaSystem. The data were subsequently analyzed by quantifying descriptive statistics (mean values, SD and SE).

### TIF assay

For the TIF assay, cells were co-stained with γH2AX and telomere *in situ* hybridization using a telomere-specific peptide nucleic acid probe. Polylysine-coated slides on which cells were grown were rinsed in PBS and fixed in 4% formaldehyde for 15 min. Cells were permeabilized in 0.2% (v/v) Triton-X in distilled water at 4.0°C for 10 min and blocked with 0.5% (w/v) bovine serum albumin (BSA) in PBS for 30 min. Anti-phospho- histone H2AX (Ser139) (Millipore, USA) was diluted 1:500 with 0.5% BSA; 100 μL of the resulting solution was added to each slide and incubated for 1 h in a humidified container. After three washes in sterile tris-buffered saline containing tween-20 (TBS-T, pH7.4; 0.15 M NaCl, 0.268 mM KCl, 0.025 M tris-base, 500 μL/1 l tween-20) for 3 min, slides were incubated with 100 μL of anti-goat secondary antibody conjugated to fluorescein isothiocyanate (FITC) and washed as described above. The slides were then placed in 4% formaldehyde for 20 min to facilitate cross-fixing and antibody preservation. The following step involved hybridization to a telomeric PNA probe, which was performed as described above with the exception that TBS-T was used for washing instead of PBS. A large number of cells (100) were analyzed for each sample. TIFs were measured before irradiation (baseline state) and at different times after exposure to ionizing radiation (30 min, 2 h, 5 h and 24 h) and 24 h after DEB treatment.

### Apoptosis assay

Blood cells: For apoptosis assay, aliquots of 0.5 ml peripheral blood from each subject were cultured in duplicate. One of these cultures was used for analysis of spontaneous apoptosis, while the second culture was set up by using the irradiated whole blood. Blood cells were incubated in medium RPMI-1640 supplemented with 15% of calf serum without phytohemaglutinin (PHA) in CO_2_ incubator for 24 h. After incubation, cells were gently washed with physiological saline (0.9% NaCl) at 37°C, fixed in methanol: acetic acid (3:1) and subsequently fixed in 96% ethanol.

Fibroblasts: At each time point after irradiation and/or DEB treatment cells were detached from the flasks surface with 0.025 M trypsin-EDTA (Gibco, Invitrogen Ltd., Paisley, UK), washed with pre-warmed PBS (Bioatlas, Tartu, Estonia) at 37°C and fixed in 96% ethanol. Apoptosis was assessed by flow cytometric (Becton Dickinson, Heidelberg, Germany) identification of cells displaying apoptosis associated DNA condensation. DNA content was assessed by measuring the UV fluorescence of propidium iodide-stained DNA. Apoptotic population analysis was performed using CellQuest software (Becton Dickinson, Franklin Lakes, NJ, USA).

### Isolation of RNA and quantitative real-time reverse transcriptase-PCR

Total RNA was isolated from each cell culture flask (both untreated fibroblasts and those treated with DEB or radiation) using the TRI reagent (Ambion, Inc.) in accordance with the manufacturer’s instructions. The quantity purified mRNA was assessed with a NanoDrop® ND-1000 spectrophotometer (Thermo Scientific, Wilmington, Delaware). Structural RNA integrity was confirmed by formaldehyde gel electrophoresis. One microgram of RNA was treated with DNAse I (Fermentas, Lithuania) and reverse transcription was performed using a first strand cDNA synthesis kit with oligo-dT18 primers (Fermentas, Lithuania) according to the manufacturer’s instructions. Mock reactions lacking reverse transcriptase (RT) were performed during the cDNA synthesis step to exclude genomic contamination. Real-time PCR was performed in duplicate in an ABI Real-time 7500 system (ABI, Foster City, CA). Detection of TRF-1 and TRF-2 gene expression was accomplished with 0.2 μmol/L of each primer and 0.1 μmol/L of probe in TaqMan® gene expression master mix in a total volume of 25 μL. The sequences of the primers and probes are as follows: TRF1, forward primer 5’-CCACATGATGGAGAAAATTAAGAGTTAT-3’, reverse primer 5’-TGCCGCTGCCTTCA TTAGA-3’, probe 5’-FAM-TTATGTGCTAAGTGAAAAATCATCAACCT-TAMRA-3’ [52]. The results from the RT-PCR assays were validated by amplifying the serial dilutions of a sample by 10-fold, and the slopes of amplification curves were calculated. Detection of internal reference 18 s rRNA was performed with pre-developed TaqMan® Gene Expression Assays ID Hs99999901_s1 (ABI, Foster City, CA), although the glyceraldehyde-3- phosphate dehydrogenase (GAPDH, Hs99999905_m1) and cyclophilin A (CYCA, Hs99999904_m1) were run on all the samples as two additional endogenous controls. Differences in mRNA expression, according to genotypes of the investigated polymorphisms, were tested with REST 09 software (Corbett Life Science, http://rest.gene-quantification.info) [53].

### Statistics

The data were analyzed using statistical software package 7.0. Statistical analyses were performed *via* Student’s *t* test. Correlations between investigated parameters were tested with linear regression analysis. A P value less than 0.05 was considered statistically significant. Differences in mRNA expression between groups were tested by pairwise randomization and boot strapping with the relative expression software tool, REST 09 (Corbett Life Science) [53].

## Competing interests

The authors declare that they have no competing interests.

## Authors’ contribution

GJ and PS designed the study. IJ, AL, SP, MO, JP, MZ and AS carried out experiments. DV and MG-S provided clinical material and helped with the analysis. JS, PS and GJ supervised experimental work in their laboratories. GJ and PS wrote the manuscript. All authors approved the final version of the manuscript.
